# Complete genome sequences of two *Pantoea stewartii* strains ATCC 8199 from maize and PSCN1 from sugarcane

**DOI:** 10.1186/s12863-024-01268-0

**Published:** 2024-10-08

**Authors:** Na Chu, Tian-Tian Liu, Hui-Li Zhang, Dong Cui, Mei-Ting Huang, Hua-Ying Fu, Jun-Bo Su, San-Ji Gao

**Affiliations:** 1 National Engineering Research Center for Sugarcane, Fujian Agriculture and Forestry University, Fuzhou, 350002 China; 2 South Subtropical Crops Research Institute, Chinese Academy of Tropical Agricultural Science, Zhanjiang, 524091 China

**Keywords:** *Pantoea stewartia*, Stewart’s bacterial wilt, Complete genome, Sugarcane (*Saccharum* spp.)

## Abstract

**Objectives:**

The pathogen of *Pantoea stewartii* (*Ps*) is the causal agent of bacterial disease in corn and various graminaceous plants. *Ps* has two subspecies, *Pantoea stewartii* subsp. *stewartia* (*Pss*) and *Pantoea stewartii* subsp. *indologenes* (*Psi*). This study presents two complete genomes of *Ps* strains including ATCC 8199 isolated from maize and PSCN1 causing bacterial wilt in sugarcane. The two bacterial genomes information will be helpful for taxonomy analysis in this genus *Pantoea* at whole-genome levels and accurately discriminated the two subspecies of *Pss* and *Psi*.

**Data description:**

The reference strain ATCC 8199 isolated from maize was purchased from Beijing Biobw Biotechnology Co., Ltd. (China) and the strain of PSCN1 was isolated from sugarcane cultivar YZ08-1095 in Zhanjiang, Guangdong province of China. Two complete genomes were sequenced using Illumina Hiseq (second-generation) and Oxford Nanopore (third-generation) platforms. The genome of the strain ATCC 8199 comprised of 4.78 Mb with an average GC content of 54.03%, along with five plasmids, encoding a total of 4,846 gene with an average gene length of 827 bp. The genome of PSCN1 comprised of 5.03 Mb with an average GC content of 53.78%, along with two plasmids, encoding a total of 4,725 gene with an average gene length of 913 bp. The bacterial pan-genome analysis highlighted the strain ATCC 8199 was clustered into a subgroup with a *Pss* strain CCUG 26,359 from USA, while the strain PSCN1 was clustered into another subgroup with a *Ps* strain NRRLB-133 from USA. These findings will serve as a useful resource for further analyses of the evolution of *Ps* strains and corresponding disease epidemiology worldwide.

**Supplementary Information:**

The online version contains supplementary material available at 10.1186/s12863-024-01268-0.

## Objective

The genus *Pantoea* includes 19 species and three uncertain species (*P*. *endophytica*, *P*. *latae*, *P*. *mediterraneensis*, and *P*. *persica*), which occurs in association with plant and animal hosts, and environmental samples, including soil and rivers [1]. Moreover, Mergaert et al. (1993) proposed that *P. stewartia* (*Ps*) is constituted by two subspecies, *P. stewartii* subsp. *stewartii* (*Pss*) and *P. stewartii* subsp. *indologenes* (*Psi*) [2]. The strains from *P. stewartia* infect various *Poaceae* plants, including leek, onion, chive, Japanese bunching onion, rice, corn, common wheat, sugarcane, foxtail millet, pearl millet, oat, lucky bamboo, and so on. The *Pss* is the agent of Stewart’s vascular wilt in corn and bacterial wilt in sugarcane [3, 4].

A PSCN1 strain was isolated from the cultivar YZ08-1095 in 2017 and this strain forms yellow-colored colonies on solid nutrient agar (NA) medium and straight rods and nonencapsulated cells were observed under transmission electron microscopy [3]. Additionally, the pathogenicity of this bacterial strain has been verified by the Koch’s postulate [3]. The strain PSCN1 was proposed as the *Pss* based on the phylogenetic analysis of bacterial 16S rDNA sequences [3]. To further exploring taxonomic classification on PSCN1, complete genome sequences of this pathogen together with the reference strain of *Pss* ATCC 8199 (provided by Beijing Biobw Biotechnology Co., Ltd., China) hosted in corn were sequenced and assembly based on the combination of the Illumina Hiseq and Oxford Nanopore platforms. Although the genome sequence derived from strain CCUG 26,359 (= ATCC 8199) has been assembled at the contig level by the Illumina MiSeq platform, this contig-level genome assembly includes 352 contigs and no assembled chromosomes (NCBI dataset: PRJNA563568). Thus, the two whole genome sequences will enable us to illustrate more accurate taxonomic classification of these pathogens at a pan-genome level. Additionally, our data provide some reference value for the prevention and control of bacterial wilt in corn and sugarcane.

### Data description

A pure culture of two strains (ATCC 8199 and PSCN1) were grown in liquid nutrient broth (NB) medium with constant shaking at 200 rpm and 28 °C for 24 h. After extraction of bacterial genomic DNA, the 10-Kb DNA library was constructed using the SQK-LSK109 linker kit according to the manufacturer’s instructions, and then sequenced by Oxford Nanopore PromethION (third-generation). Meanwhile, a 350-bp library was constructed using another sequence platform of Illumina Hiseq (second-generation). A total of 1,325 Mb and 1,915 Mb Nanopore clean data were generated in two strains ATCC 8199 and PSCN1 with an estimated 276.73× and 380.43× average depth of sequencing coverage, respectively (Table 1, Data file1) [5]. After quality control of the sequencing data, subreads from the Nanopore platform were assembled with Canu (version 1.5) [21]. The assembly results were further corrected with Illumina data using Pilon [22]. The genome of ATCC 8199 strain is 4.78 Mb in total with GC content of 54.03%, including one circular chromosome (4,526,106 bp) and five plasmids (P1–P5, with 25,199, 35,601, 62,815, 65,465, and 73,276 bp, respectively). The genome PSCN1 strain is 5.03 Mb in total with GC content of 53.78%, containing one circular chromosome (4,511,897 bp) and two plasmids (pCN101- pCN102 with 310,867 and 211,928 bp, respectively) (Data file1) [5]. Gene prediction identified 4,846 genes in ATCC 8199 strain and 4,725 genes in PSCN1 strain using Prodigal [23]. The genome of ATCC 8199 strain includes 71 tRNAs, 21 rRNAs, 6 ncRNA, 11 CRISPR numbers, 17 genomic islands, and 4 prophages. Genomic component analysis revealed that PSCN1 contained 78 tRNAs, 22 rRNAs, 18 ncRNA, 8 CRISPR numbers, 17 genomic islands, and one prophage (Data file1) [5]. The complete genome sequences of PSCN1 and ATCC 8199 have been deposited in GenBank dataset under the accession numbers CP046585-CP046587 and CP046558-CP046563, respectively.

**Table 1:** Overview of data files/data sets

Label	Name of datafile/data set	File types (file extension)	Data repository and identifier (DOI or accession number)
Data file 1	General features of two genomes from PSCN1 and ATCC 8199 strains	Word file (.docx)	Figshare (10.6084/m9.figshare.26166673) [5]
Data file 2	Genome organization and gene distribution in two strain PSCN1(A) and ATCC 8199(B)	Word file (.docx)	Figshare (10.6084/m9.figshare.26170231) [6]
Data file 3	Summary of gene annotation of the strain ATCC 8199	Excel file (.xlsx)	Figshare (10.6084/m9.figshare.26170141) [7]
Data file 4	Summary of gene annotation of the strain PSCN1	Excel file (.xlsx)	Figshare (10.6084/m9.figshare.26170150) [8]
Data file 5	Characteristics of strains used in this study	Excel file (.xlsx)	Figshare (10.6084/m9.figshare.26170171) [9]
Data file 6	Average nucleotide identity based on the entire genome sequence of 47 strains of *Pantoea stewartia*. and one strain of *Pantoea agglomerans*	Excel file (.xlsx)	Figshare (10.6084/m9.figshare.26170210) [10]
Data file 7	Phylogeny tree analysised with core-gene sequence of 42 strains of *Pantoea stewartii* and one strain of *Pantoea agglomerans*	Word file (.docx)	Figshare (https://doi.org/10.6084/m9.figshare.26170219) [11]
Dataset 8	Genome assembly of *Pantoea stewartia* subsp. *stewartia* strain ATCC 8199 chromosome.	GenBank file (.bp)	GenBank (https://identifiers.org/ncbi/insdc:CP046558) [12]
Dataset 9	Genome assembly of *Pantoea stewartia* subsp. *stewartia* strain ATCC 8199 plasmid p1.	GenBank file (.bp)	GenBank (https://identifiers.org/ncbi/insdc:CP046559) [13]
Dataset 10	Genome assembly of *Pantoea stewartia* subsp. *stewartia* strain ATCC 8199 plasmid p2.	GenBank file (.bp)	GenBank (https://identifiers.org/ncbi/insdc:CP046560) [14]
Dataset 11	Genome assembly of *Pantoea stewartia* subsp. *stewartia* strain ATCC 8199 plasmid p3.	GenBank file (.bp)	GenBank (https://identifiers.org/ncbi/insdc:CP046561) [15]
Dataset 12	Genome assembly of *Pantoea stewartia* subsp. *stewartia* strain ATCC 8199 plasmid p4.	GenBank file (.bp)	GenBank (https://identifiers.org/ncbi/insdc:CP046562) [16]
Dataset 13	Genome assembly of *Pantoea stewartia* subsp. *stewartia* strain ATCC 8199 plasmid p5.	GenBank file (.bp)	GenBank (https://identifiers.org/ncbi/insdc:CP046563) [17]
Dataset 14	Genome assembly of *Pantoea stewartia* strain PSCN1 chromosome.	GenBank file (.bp)	GenBank (https://identifiers.org/ncbi/insdc:CP046585) [18]
Dataset 15	Genome assembly of *Pantoea stewartia* strain PSCN1 plasmid pCN101.	GenBank file (.bp)	GenBank (https://identifiers.org/ncbi/insdc:CP046586) [19]
Dataset 16	Genome assembly of *Pantoea stewartia* strain PSCN1 plasmid pCN102.	GenBank file (.bp)	GenBank (https://identifiers.org/ncbi/insdc:CP046587) [20]

Gene annotation was determined with the BLAST program [24] and with 12 different databases. The overview of the two complete genomes were presented to the annotation information using Circos [25] (Data file2) [6]. For the ATCC 8199 strain, GO analysis [26] revealed that 3,337 genes were assigned into 41 GO categories, with the most genes in catalytic activity (2,090 genes). KEGG analysis [27] revealed that 2,612 genes were significantly enriched in 106 pathways. A total of 559 putative virulence factors, 797 pathogen–host interaction genes, 404 transport proteins, 144 carbohydrate active enzymes, and 3 antibiotic resistance proteins were annotated based on the VFDB database [28], PHI-base [29], TCDB database [30], CAZyme database [31] and ARDB database [32], respectively (Data file3) [7]. For the PSCN1 strain, 2,790 genes were assigned into 41 GO categories. The largest category was assigned to catalytic activity (1,772 genes). A total of 2785 genes were significantly enriched in 107 KEGG pathways. A total of 695 putative virulence factors, 911 pathogen–host interaction genes, 418 transport proteins, 164 carbohydrate active enzymes, and 3 antibiotic resistance proteins were annotated based on those above-mentioned datasets (Data file4) [8].

The two genome sequences obtained in this study along with 45 *Ps* strains retrieved from NCBI library were used for sequence analysis. A strain of *P. agglomerans* CFSAN047153 was used an outgroup (Data file5) [9]. The average nucleotide identities (ANI) were calculated by pairwise genome comparison based on BLAST+ and FastANI [33]. The 47 *Ps* strains worldwide shared 98.40–99.99% ANI indexes and shared 80.89–81.38% ANI index with the strain CFSAN047153 of *P. agglomerans*. The two strains ATCC 8199 and PSCN1 had 98.56% ANI index with each other and shared 98.41-99.97% and 98.30-99.07% with other *Ps* strains, respectively (Data file6) [10]. Notably, ATCC 8199 and PSCN1 had highest ANI indexes with IPV-BO 2766 (NCBI dataset: PRJNA856801) and HR3-48 (NCBI dataset: PRJNA844595) strains, respectively.

To exploring the phylogenetic relationship between PSCN1 and ATCC 8199 with other strains, the gene family clustering was carried out based on the alignment of single copy genes identified with OrthoMCL [34]. The phylogenetic tree was constructed with core-gene sequence of 43 *Pantoea* strains using maximum likelihood method and 1,000 bootstrap replications using PhyML [35]. Ten *Ps s*trains including ATCC 8199 and PSCN1 were clustered into one subclade, which would be further separated into three groups. Furthermore, the strains HR3-48 (*Ps*) and LMG 2671 (*Psi*) were clustered together in the group (I) The strains PSCN1 and NRRLB-133 (*Ps*) were clustered together in the group (II). Other six *Pss* strains including ATCC 8199 were clustered in the group III (Data file7) [11]. The PSCN1 was proposed as a *Pss* strain based on bacterial 16S rDNA sequences [3], but this pathogen might be a *Psi* strain at pan-genome level. However, an accurate classification of two subspecie*s Pss* and *Psi* within this genus *Pantoea* need be further confirmed based on numerous complete genome sequences and biological experiments.

### Limitations

This data note presented two complete genome sequences, one from reference strain ATCC 8199 hosted in corn and another strain PSCN1 isolated from infected sugarcane plants showing bacterial wilt symptoms. However, only a single strain genome sequence from sugarcane in China. More strains need be collected at a global context and used for the whole genome sequencing. Thus, taxonomic classification of this bacterial species would be further accurately illustrated.

## Electronic supplementary material

Below is the link to the electronic supplementary material.

Supplementary Material 1

Supplementary Material 2

Supplementary Material 3

Supplementary Material 4

Supplementary Material 5

Supplementary Material 6

Supplementary Material 7

## Data Availability

The genome assembly data that support the findings of this study have been deposited in NCBI GenBank under the accession numbers CP046558-CP046563 and CP046585-CP046587 (Table 1).

## Abbreviations

ANI: Average Nucleotide Identity
Bp: Base pair
BLAST: Basic Local Alignment Search Tool
NCBI: National Center for Biotechnology Information
NB: Nutrient broth
