# Supplementary material for: Complete genome sequences of two Pantoea stewartii strains ATCC 8199 from maize and PSCN1 from sugarcane
Source: BMC Genom Data. 2024 Oct 8;25:86. doi: 10.1186/s12863-024-01268-0 (PMC11462666; doi:10.1186/s12863-024-01268-0)
Supplement: Supplementary file 1 — Supplementary Material 1 [file 12863_2024_1268_MOESM1_ESM.docx]

**Supplementary data file 1**

**Table S1.** General features of two genomes from PSCN1 and ATCC 8199 strains

| Element and characteristics | PSCN1 | ATCC 8199 |
| --- | --- | --- |
| Coverage | 380.43× | 276.73× |
| Conting number | 3 | 6 |
| Size (Mb) | 5.03 | 4.78 |
| N50 | 4,511,897 | 4,526,106 |
| G + C content (%) | 53.78 | 54.03 |
| plasmid | 2 | 5 |
| Gene number | 4725 | 4846 |
| tRNA | 78 | 71 |
| rRNA | 22 | 21 |
| ncRNA | 18 | 6 |
| CRISPR numbers | 8 | 11 |
| Genomic islands | 17 | 17 |
| Prophage | 1 | 4 |
