# Supplementary material for: Complete genome sequences of two Pantoea stewartii strains ATCC 8199 from maize and PSCN1 from sugarcane
Source: BMC Genom Data. 2024 Oct 8;25:86. doi: 10.1186/s12863-024-01268-0 (PMC11462666; doi:10.1186/s12863-024-01268-0)
Supplement: Supplementary file 2 — Supplementary Material 2 [file 12863_2024_1268_MOESM2_ESM.docx]

**Supplementary data file 2**


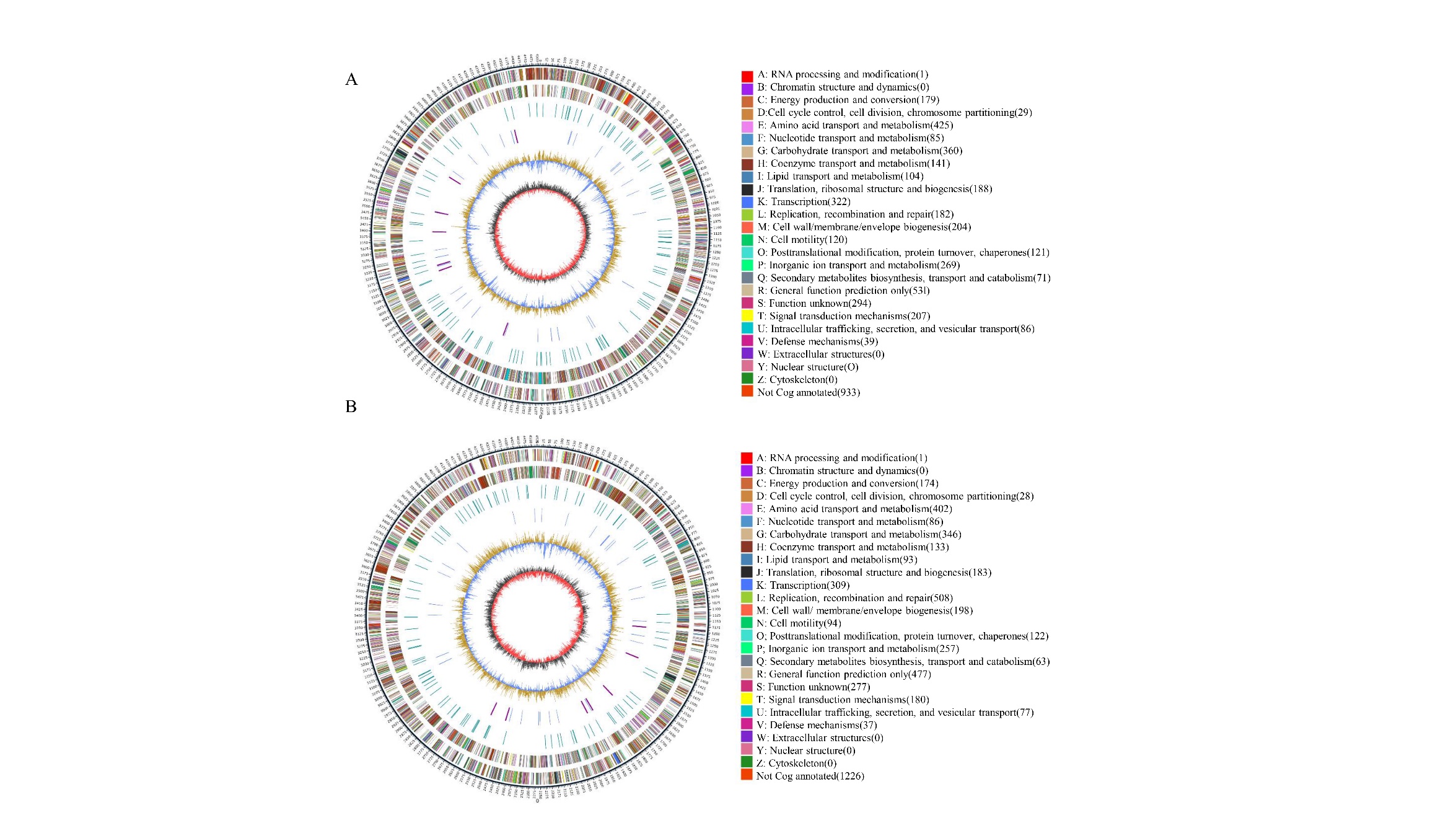


**Figure S1. Genome organization and gene distribution in two strain PSCN1 (A) and ATCC 8199 (B).** The outermost circle is a marker of genome size, with each scale measuring 5 kb; The second and third circles represent genes on the positive and negative strands of the genome, respectively, with different colors representing different COG functional classifications; The fourth circle is a repetitive sequence; The fifth circle consists of tRNA and rRNA, with blue representing tRNA and purple representing rRNA; The sixth circle represents the GC content. The light yellow part indicates that the GC content in this region is higher than the average GC content of the genome. The higher the peak, the greater the difference between the GC content and the average GC content. The blue part indicates that the GC content in this region is lower than the average GC content of the genome; The innermost circle is GC skew, with dark gray representing areas where G content is greater than C, and red representing areas where C content is greater than G.
