# Supplementary material for: Complete genome sequences of two Pantoea stewartii strains ATCC 8199 from maize and PSCN1 from sugarcane
Source: BMC Genom Data. 2024 Oct 8;25:86. doi: 10.1186/s12863-024-01268-0 (PMC11462666; doi:10.1186/s12863-024-01268-0)
Supplement: Supplementary file 7 — Supplementary Material 7 [file 12863_2024_1268_MOESM7_ESM.pptx]

## Slide 1
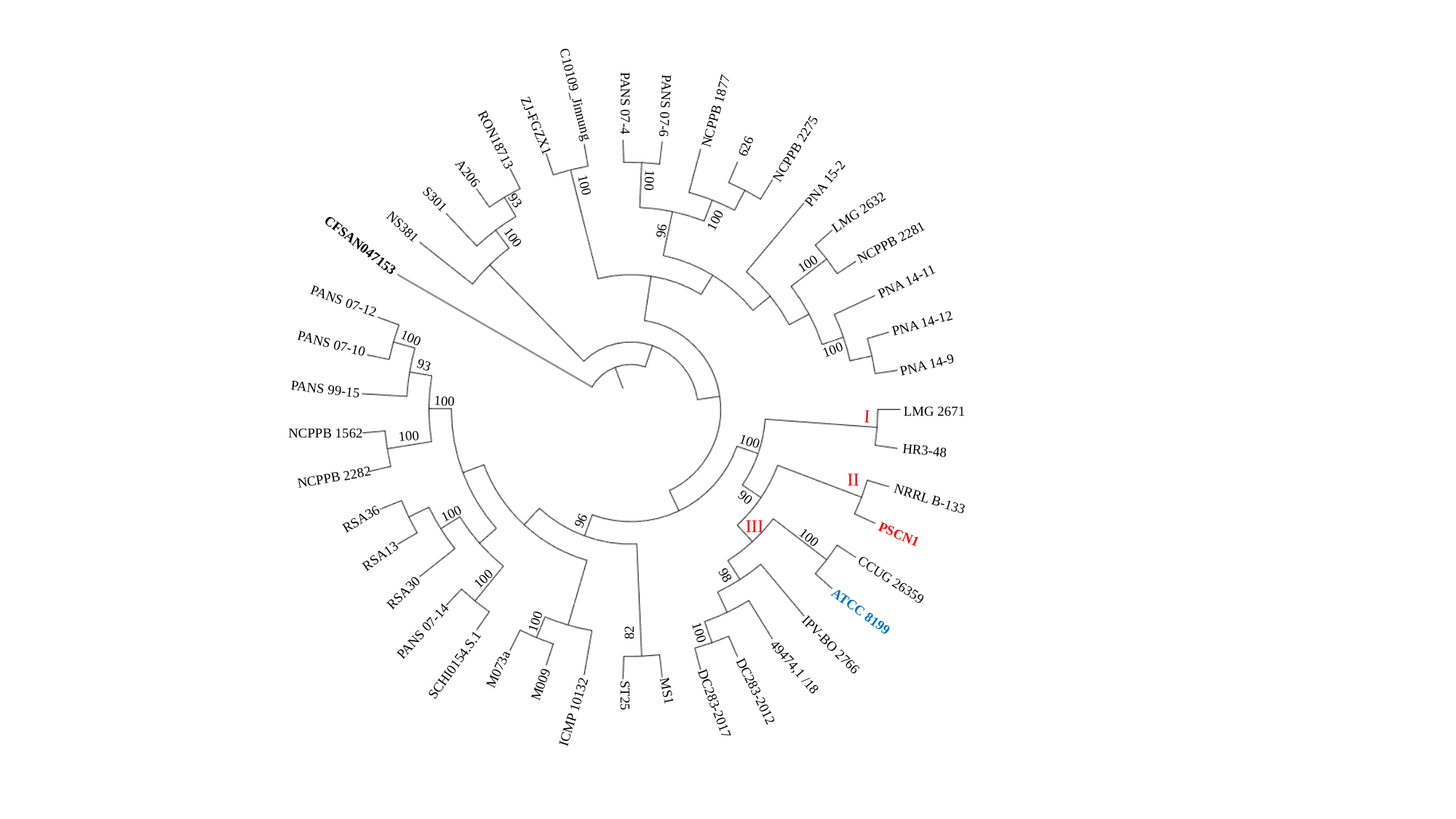

C10109_Jinnung
PANS 07-4
PANS 07-6
NCPPB 1877
ZJ-FGZX1
RON18713
626
NCPPB 2275
A206
PNA 15-2
S301
LMG 2632
NS381
NCPPB 2281
CFSAN047153
PNA 14-11
PANS 07-12
PNA 14-12
PANS 07-10
PNA 14-9
PANS 99-15
LMG 2671
NCPPB 1562
HR3-48
NCPPB 2282
NRRL B-133
RSA36
PSCN1
RSA13
CCUG 26359
RSA30
ATCC 8199
PANS 07-14
IPV-BO 2766
SCHI0154.S.1
49474,1 /18
M073a
M009
DC283-2012
MS1
ST25
DC283-2017
ICMP 10132
100
100
93
100
96
100
100
100
100
93
100
100
100
90
100
96
100
100
98
100
82
100
I
 II
III

## Slide 2
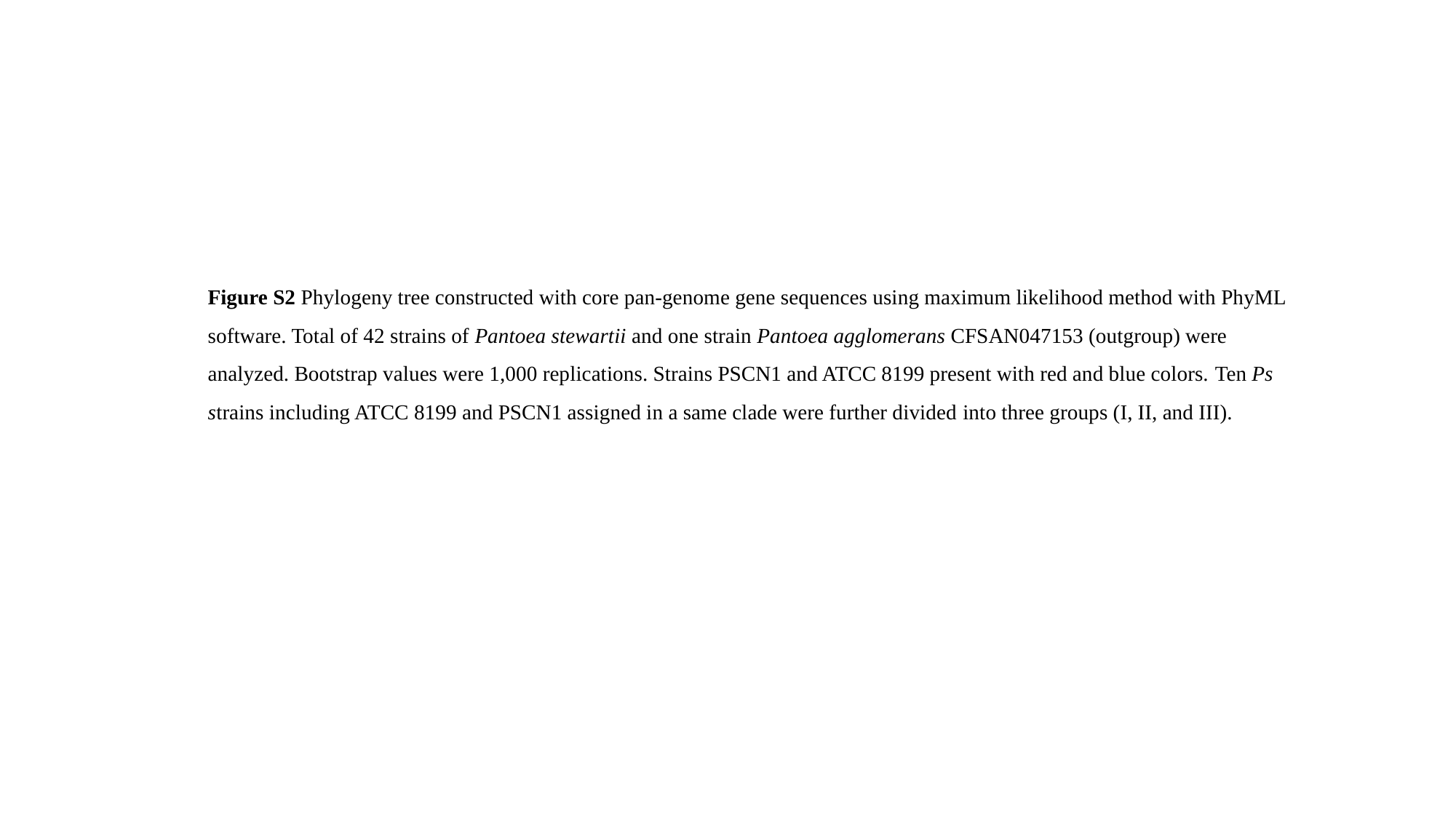

Figure S2 Phylogeny tree constructed with core pan-genome gene sequences using maximum likelihood method with PhyML software. Total of 42 strains of Pantoea stewartii and one strain Pantoea agglomerans CFSAN047153 (outgroup) were analyzed. Bootstrap values were 1,000 replications. Strains PSCN1 and ATCC 8199 present with red and blue colors. Ten Ps strains including ATCC 8199 and PSCN1 assigned in a same clade were further divided into three groups (I, II, and III).
